# Exploring Spatial Correlations of Tourism Ecological Security in China: A Perspective from Social Network Analysis

**DOI:** 10.3390/ijerph20053912

**Published:** 2023-02-22

**Authors:** Zhaofeng Wang, Dongchun Huang, Jing Wang

**Affiliations:** Tourism College, Hunan Normal University, Changsha 410081, China

**Keywords:** tourism ecological security, DPSIR model, spatial network structure, quadratic assignment procedure, influencing factors

## Abstract

The imbalance of regional tourism ecological security (TES) is an important barrier to the sustainable development of tourism. Relying on the spatial correlation network to coordinate the regional TES is effective. Taking 31 provinces in China as examples, social network analysis (SNA) and the quadratic assignment procedure (QAP) are used to analyze the spatial network structure of TES and its influencing factors. The results show that (1) the network density and the number of network relationships increased, while the network efficiency remained at approximately 0.7, and the network hierarchy decreased from 0.376 to 0.234. (2) Jiangsu, Guangdong, Shandong, Zhejiang, and Henan were always more central than the average and dominated. Anhui, Shanghai, and Guangxi have much lower centrality degrees than the average, with little effect on other provinces. (3) The TES networks could be divided into four parts: “net spillover”, “agent”, “bidirectional spillover” and “net benefit”. (4) The differences in economic development level, tourism industry dependence, tourism load level, educational attainment, investment in environmental governance, and transportation accessibility all had a negative impact on the TES spatial network, whereas geographic proximity had a positive driving effect. In conclusion, the spatial correlation network of provincial TES in China is increasingly close, but the network structure is loose and hierarchical. The core–edge structure is obvious, and there are significant spatial autocorrelations and spatial spillover effects between provinces. The difference in regional influencing factors has a significant effect on the TES network. This paper presents a new research framework for the spatial correlation of TES and provides a Chinese solution to promote the sustainable development of tourism.

## 1. Introduction

Tourism ecological security (TES) ensures that the resources that tourism depends on are in a sustainable and healthy balance state, which is an important evaluation index of the sustainable development of tourism [1]. The disparity in tourism resource endowment and the aggregation of economic and social activities across regions result in a serious imbalance in regional TES [2]. With the advancement of market integration and changes in transportation technology, elements of TES, such as capital, energy, information, and talents, have realized a trans-regional flow [3], which makes the spatial connection range of TES broader. Thus, relying on the spatial correlation network provides a new strategy for coordinating the regional TES and promoting the sustainable development of tourism. Tourism is a driving factor in supporting economic and social growth and improving people’s lives in China, the world’s largest developing country. However, with the continuous growth of the tourism economy, China’s tourism resources have long been in a state of excess consumption. As a result, the tourism destination ecosystem has been greatly harmed, and an imbalance in inter-provincial TES has emerged, which has gradually become a key issue limiting China’s sustainable tourism development [2,4]. Therefore, taking 31 provinces in China as an example, the exploration of the TES spatial network structure and its influencing factors is expected to provide a Chinese solution for other countries to coordinate the sustainable development of regional tourism.

TES is closely related to ecosystem theory and ecological security theory [1]. TES could be defined as a state in which the tourism ecosystem maintains structural stability and functional diversity, provides an abundance of material foundation and environmental space for tourism development, and maintains the coordination and balance of the complex ecosystem composed of the economy, society, and nature in the tourism destination [4,5]. Many scholars have conducted empirical studies on TES, focusing primarily on the following aspects. For index system construction, the index evaluation model is the most widely used in the literature and mainly includes the PSR [6], PSR-EES [7], and DPSIR [2,8,9] models. In terms of quantitative methods, the commonly used quantification methods are as follows: the comprehensive index method [10], ecological footprint method [11], grey relation projection method [12], improved TOPSIS method [13], and entropy weight TOPSIS method [6]. Regarding the research content, foreign countries have mostly focused on the impact of tourism on the ecological environment [14], the relationship between the tourism environment and society [15], tourism carrying capacity [16,17], and so on. In China, there are many studies directly related to TES [17]. Some scholars have made continuous attempts regarding the assessment [6,18], spatiotemporal patterns [1,2,3], driving mechanisms [1], and prediction [6,19]. It is worth noting that the study of spatial correlation in tourism geography is an essential consideration of TES. The literature has explored the spatial characteristics of TES in many regions, and significant spatial autocorrelation has been observed [20,21]. The spatial econometric model was used by Li et al. [4] and Ma et al. [3], who discovered a significant spatial spillover effect on inter-regional tourism ecological security. 

The existing literature focuses primarily on the spatial characteristics of TES based on attribute data, with only a few studies on the spatial correlation of TES based on relational data. However, structure frequently determines the performance of attribute data, which is of great analytical significance [22]. Furthermore, spatial econometric models have become a popular method for studying TES spatial correlation. This model, however, is based on attribute data, and its research scope is limited to geographically adjacent areas, making it difficult to understand the overall spatial correlation. 

To fill the above gap, we propose a research hypothesis that the spatial correlation of TES presents a complex spatial network structure between provinces. Therefore, this paper constructs the TES evaluation index system according to the DPSIR model framework. Then, the entropy weight TOPSIS method is used to calculate the TES index of 31 provinces in China from 2005 to 2020. On this basis, a spatial correlation network based on a modified gravity model is constructed. Then, SNA and QAP are used to analyze the spatial network structure of TES and its influencing factors in China. Finally, policy suggestions are proposed to coordinate regional TES development. With this background, we have conducted the present study with the following objectives: (i) grasp the overall spatial correlation of TES; (ii) enrich and expand the influence mechanism of the TES network; (iii) provide Chinese solutions and a new research framework to coordinate the development of regional TES for other parts of the world.

**Figure 1:**
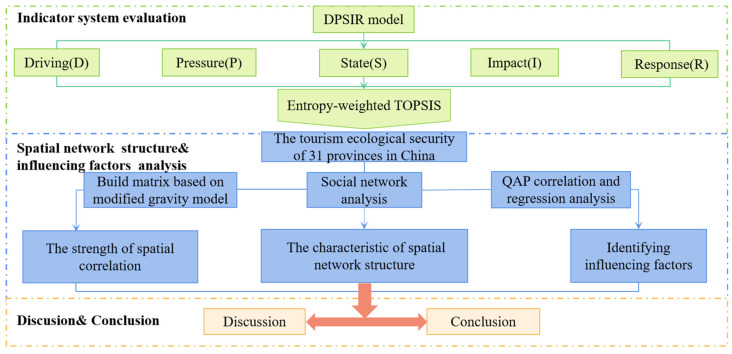
A spatial network research framework for TES.

The remainder of the study is organized as follows: Section 2 introduces methodologies and data sources. Section 3 examines the spatial network structure of TES and its influencing elements. Section 4 focuses on the research findings and limitations. Section 5 focuses on theoretical implications and practical implications. To show the research ideas more clearly, a chart of the paper framework is drawn as follows (Figure 1).

## 2. Methodology and Data Source

### 2.1. Methodology

#### 2.1.1. Entropy Weight TOPSIS Method

The entropy weight TOPSIS method is a combination of the entropy weight and TOPSIS methods. Weight allocation based on entropy can effectively reduce the impact of traditional subjective weighting methods on the effectiveness of the evaluation results [23,24,25]. Meanwhile, TOPSIS method is a common and effective multi-objective system decision-making method with no strict constraints on data distribution, sample size, or index number. This method can be used for horizontal multi-indicator comparison and longitudinal analysis in different years, and it is applicable not only to small data samples but also to multiple evaluation objects and indicators [26]. It has been successfully used by many scholars in the research of land use [27], land ecological security [28], and tourism ecological security [21,29,30]. The main procedure is to allocate the weight based on entropy, and then the TOPSIS method is used to calculate the closeness degree between each scheme and the ideal solution. The value of the closeness degree is between 0 and 1. The higher the value, the more secure the tourism ecosystem will be; otherwise, it will be worse. The specific calculation formulas are based on the literature [13].

(1) $$C_{i}=\frac{d_{i}^{-}}{d_{i}^{+}+d_{i}^{-}}\left(i=1,2,\ldots ,m\right)$$

(2) $$d_{i}^{+}=\sqrt{\sum_{j=1}^{n}{\left(a_{ij}-a_{j}^{+}\right)}^{2}};d_{i}^{-}=\sqrt{\sum_{j=1}^{n}{\left(a_{ij}-a_{j}^{-}\right)}^{2}}\left(i=1,2,\ldots ,m\right)$$

where $a_{ij}$ denotes the indicator after multiplying the standardized indicator by the weights; $a_{j}^{+}$ denotes the maximum value of indicator j, while $a_{j}^{-}$ denotes the minimum value of indicator j.

#### 2.1.2. DPSIR Model

The DPSIR model was developed by the European Environment Agency to solve the problems of the environment and resource management. It is not only a common model that objectively reflects the interaction and influence between tourism activities and the ecological environment but can also scientifically explain the autonomous and positive feedback mechanism of human society [1]. The DPSIR in the TES system is an orderly and sustainable operating and open circular system, and its operating mechanism is as follows: social and economic factors are the continuous driving force affecting tourism ecosystem operation (D). In the process of tourism development, extensive economic growth, environmental capacity overload and excessive waste discharge will cause great pressure on the ecological environment and human society (P). The pressure gradually affects the operating state of the tourism ecosystem, causing some changes in the tourism economy, social services, and environmental conditions (S). At the same time, it will also have an impact on the contribution rate of the tourism economy, the capacity of the tourism environment to break the limit, the discharge of tourism waste exceeding the standard, and the overload of tourism infrastructure (I) and encourage individuals to take meaningful action to maintain the harmonious coordination of tourism growth and the ecological environment (R). For example, fiscal expenditure should be increased, environmental pollution control efforts should be strengthened, high-quality tourism personnel should be trained, and resource recycling technology should be improved. The feedback effect generated by such a response finally has a positive impact on D, P, and S through the interaction and integration of internal elements of the system, forming a virtuous cycle of the regional TES system. Following the principles of system, integrity, and effectiveness, and based on the connotation of TES, indicators and evaluation framework are selected according to the DPSIR model to construct the following provincial TES evaluation index system in China (Table 1).

#### 2.1.3. Modified Gravity Model

The determination of relationships is not only the basis of constructing a spatial correlation network but also the key to determining the structural characteristics of the spatial correlation network of TES in China. Most existing studies use gravity model to determine the correlation between nodes, which can comprehensively consider the impact of factors including economy and distance. It is widely used in research on tourism flow [32], the tourism economy [33], and tourism efficiency [34]. By using the gravity model to establish a correlation network, TES can be combined with economic distance and geographical distance to better reveal the spatial correlation characteristics of TES. The calculation formula is as follows:

(3) $$F_{ij}=K_{ij}\frac{N_{i}N_{j}}{D_{ij}^{2}},K_{ij}=\frac{G_{i}}{G_{i}+G_{j}},D_{ij}^{2}={\left(\frac{d_{ij}}{g_{i}-g_{j}}\right)}^{2}$$

where *F_ij_* is the link strength of the TES, *K_ij_* is the gravity coefficient, *N* is the TES index, *D_ij_* is the spherical distance between province *i* and province *j*, and *G* and g represent GDP per capita and GDP, respectively. Although the dichotomous method is convenient in terms of data collection and input, image output, and a sparse study structure, the premise is that the appropriate threshold is selected. Referring to existing research [33,35], the average value of each row of data is taken as the threshold and binarized to construct the spatial correlation matrix of TES. If the strength of the connection exceeds the threshold value, the value is 1, suggesting that there is a TES connection between provinces; otherwise, the value is 0, indicating that there is no connection.

#### 2.1.4. Social Network Analysis (SNA)

With its evident benefits of clear graphics and precise data calculation, SNA is based on graph theory and algebra to evaluate the network relations among social members and the characteristics of network structure [36]. SNA converts attribute data into relational data, explores the overall spatial association of regions, and breaks through the limitations of exploring the spatial relationship between geographically adjacent regions. Therefore, based on SNA, the overall characteristics, individual characteristics, and block patterns of the spatial correlation network of TES in China are studied. The overall structural characteristics are mainly measured by the number of relationships, the density of the network, the efficiency of the network, the hierarchy of the network, and the correlation degree, which can clarify the overall situation and the overall structural characteristics of the TES network. Individual characteristics can be measured by degree centrality, betweenness centrality, and closeness centrality, which can quantify the position and power of nodes in the network and the role they play in the network. Block mode analysis mainly uses CONCOR to cluster and partition the spatial correlation network, reveal the internal structure and overflow path of the networks, and analyze the correlation characteristics within and between plates to determine the roles and positions of each plate in the spatial correlation network. The formulas of these indices refer to previous studies [33].

#### 2.1.5. Quadratic Assignment Procedure (QAP)

Since “relational data” are used to explore the association structure of China’s TES network and its influencing factors, there may be a phenomenon of “multicollinearity” among variables. However, the QAP is a nonparametric method to explore the relationship between matrices by replacing and comparing different matrix data [37]. This method does not need to assume that explanatory variables are independent of each other, so it can effectively solve the endogeneity problem of relational data [20]. Therefore, the QAP program in UCINET software is used in this paper to distinguish and extract the influencing factors of TES spatial correlation network, and fitting regression is carried out for their effect degree to avoid the deviation of results caused by the multicollinearity problem of explanatory variables.

### 2.2. Data Sources

The 31 provinces of China (excluding Hong Kong, Macao, and Taiwan) are the case in this research, and the study period runs from 2005 to 2020. The National Economic and Social Statistics Bulletin, the Provincial Statistical Yearbooks (2006–2021), the China Statistical Yearbook, the China Tourism Statistical Yearbook, the China Environmental Statistical Yearbook, and other sources provide the majority of the data (2005–2020), as well as official government websites such as provincial cultural and tourism departments. Some missing data are filled in through linear interpolation.

## 3. Results

### 3.1. Structural Characteristics of the Spatial Network

**Figure 2:**
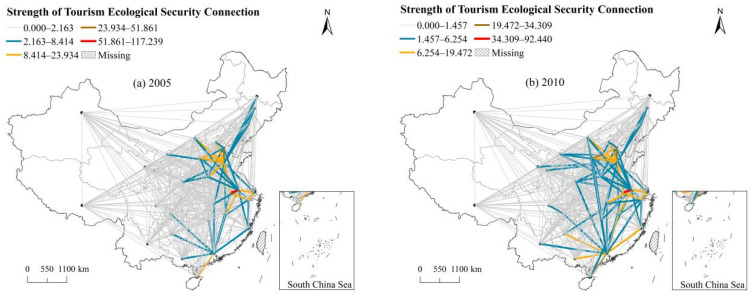

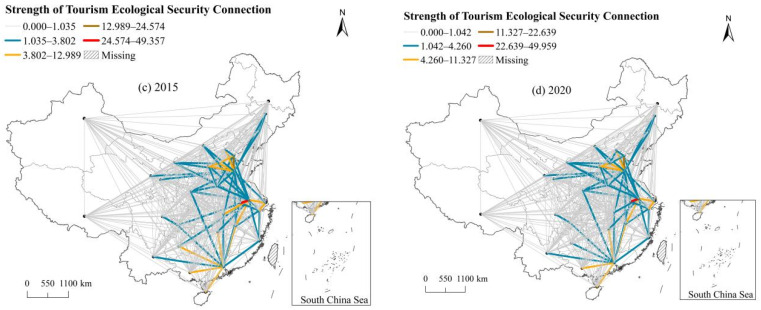
Spatial correlation network of TES in China.

The spatial connection of interprovincial TES gradually breaks through the limitation of geographical proximity, which not only produces a linkage effect on adjacent provinces but also has a spatial correlation relationship with nonadjacent provinces, resulting in complex spatial network characteristics. Meanwhile, the regional differences in strength of TES connection are obvious, generally showing the difference of “east > central > west”, which is closely related to geographical location, economic development, and transportation convenience (Figure 2). To further illustrate the spatial association network of TES, this study includes a detailed evaluation of the overall network features and individual network features.

#### 3.1.1. Overall Network Characteristics

**Figure 3:**
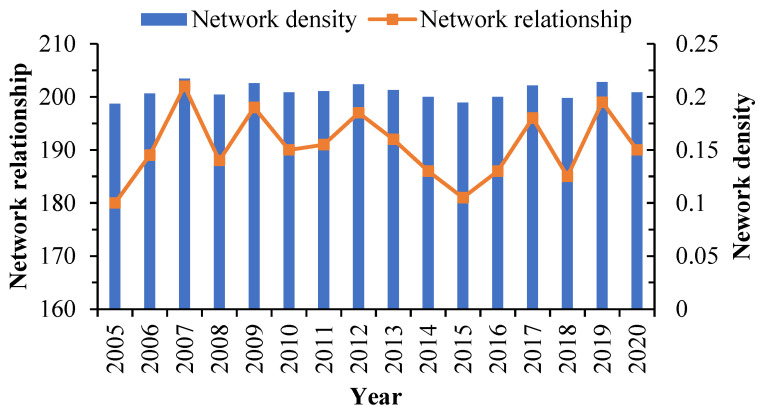
Network density and network relationships.

**Figure 4:**
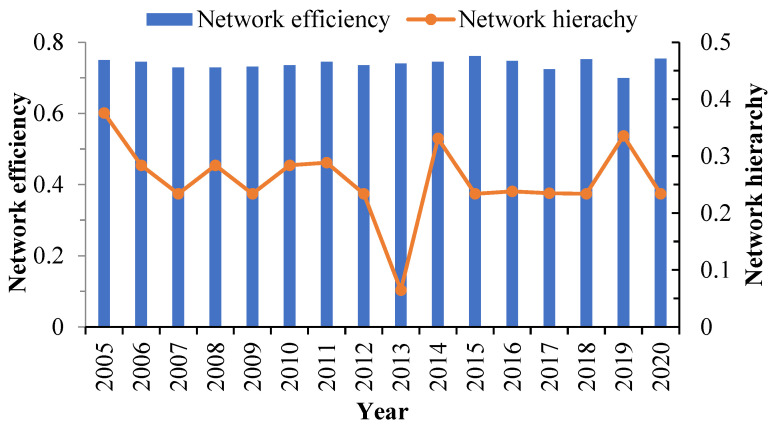
Network efficiency and network hierarchy.

The network density increased from 0.1935 to 0.2043; however, it remained much lower than the medium level of 0.5. The number of network connections grew from 180 to 190. Theoretically, the maximum number of TES network relationships ought to be 930, while its real maximum is only 202 (Figure 3). Despite the fact that the spatial association of TES is becoming stronger, the network structure is still fragile. The network efficiency is still approximately 0.738, suggesting that the TES network is well connected, but there are many overlapping spatial spillovers. The network hierarchy fluctuates and decreases, with an average of 0.257, indicating that the spillover relationship between some provinces is asymmetric to a large extent and that there is a certain degree of hierarchy (Figure 4).

#### 3.1.2. Individual Network Characteristics

The average degree centrality of TES is 29.462, with Jiangsu, Guangdong, Shandong, Zhejiang, and Henan well exceeding the mean value. These provinces’ TES is strongly linked to that of other provinces, so they are central in the spatial correlation network. These provinces, in particular, have higher out-degree point centrality and higher in-degree point centrality, suggesting that they have more spatial spillovers to other provinces and receive more spatial spillovers from other provinces. However, in general, they receive more spatial spillovers from other provinces. The provinces with a small degree of centrality include Guangxi, which is geographically located in a marginal region, and Shanghai and Anhui, which are densely populated, consume large amounts of resources, and are in the relatively marginal position of the spatial correlation network. Their in-degree point centrality and out-degree point centrality are significantly small, implying that there is no significant spatial spillover between the TES of these provinces and other provinces.

Betweenness centrality has a mean value of 2.469. The betweenness centrality of Jiangsu, Guangdong, Shandong, Zhejiang, and Henan Provinces is significantly higher than the mean value, and there is a large gap between them and other provinces. As a result, they hold the dominant control positions in the TES network and serve as bridges and ties. The betweenness centrality of Anhui, Shanghai, and Guangxi is small, close to 0. Because these provinces have limited influence on other regions inside the spatial network, they are on the periphery.

Closeness centrality has a mean value of 59.266. Jiangsu, Guangdong, Shandong, Zhejiang, and Henan Provinces have a closer centrality than the norm. They have great spatial independence from other provinces and are likely to have a direct association with other provinces. The closeness centrality of Guangxi, Shanghai, Anhui, and Hainan is low, which indicates that the TES of these regions is mainly indirectly related to other provinces when it has spatial correlation. Therefore, it cannot have an obvious driving effect on other provinces, and it is also less affected by other provinces (Table 2).

### 3.2. Cohesive Group of the Spatial Network Structure

Block model analysis is utilized to highlight the importance of every region inside the network and its interaction. To ensure that there are more than three provinces in each plate, the CONCOR algorithm is employed, with the maximum segmentation depth fixed at 2 and the convergence standard required to be 0.2 [38]. Finally, China’s thirty-one provinces are separated into four plates. There are 190 TES network relationships in all, including 33 intraplate relationships and 157 interplate relationships. Therefore, the spatial spillover effect of TES is mainly interplate spillovers, while the intraplate synergistic effect is not obvious (Table 3).

The number of external spillover ties in Plate I, 91, is far more than the number of internal interactions, 29. Furthermore, the predicted internal ratio is higher than the actual internal ratio, resulting in a considerable spatial spillover impact on adjacent plate provinces and classifying the plate as a “net spillover.” The acceptance relationships and spillover relationships of Plate II are more balanced. With its unique geographical location, Plate II plays more of a “bridge” role and can be classified as the “agent” plate. Plate III has 29 spillover relationships and 48 acceptance links, indicating that this plate not only boosts the spillover of TES elements in provinces inside the plate but also plays a spillover role in provinces beyond the plate. It is classified as the “bidirectional spillover” plate. The acceptance relations for Plate IV total 67, while the spillover relations total 14. It is clear that the TES network mostly receives spillovers from neighboring provinces, and the plate is designated as a “net benefit” plate.

To more intuitively describe the correlation between plates, the density matrix between and within the four plates in 2020 is calculated. If the average of the entire network density is larger than 0.2043, the value is 1, indicating that each plate has an association relationship; otherwise, the value is 0, indicating that no association relationship exists. As a result, the image matrix is depicted in Table 4 below.

**Figure 5:**
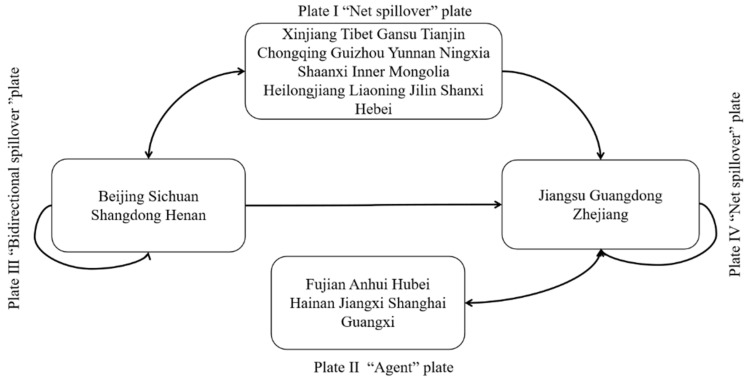
The interaction of the four plates.

Plates III and IV are not only linked to internal TES, but they are also affected by TES spillover from those other plates. Plate III, for illustration, acquires the spillover effect from Plate IV, whereas Plate IV gains the spillover effect of Plates I–III. Plates I and II have no internal TES spillover when compared to Plates III and IV. Because of a shortage of cooperation, there is a lack of rich cooperation as well as connections of TES in Plates I and II. However, Plates I and II effectively receive radiation effects from Plates III and IV, respectively. The interaction between the four plates is visualized in Figure 5.

### 3.3. Analysis of Influencing Factors

According to Pan (2012), spillover or agglomeration effects are much more likely to occur in neighboring provinces [5]. However, geographical proximity is not the only factor producing spatial correlation, and regional differences will also exert a considerable impact on the creation of spatial correlation. According to the research findings currently available [1,4,21,39], economic, transportation, resource, and environmental factors are all intimately tied to TES. Therefore, based on existing studies, the QAP model is constructed:

(4) $$TESM=f\left(W,E,L,F,J,H,T\right)$$

where *TESM* is the spatial correlation matrix of TES and *W* is the spatial adjacency matrix, with adjacency set to 1 and nonadjacency set to 0. *E*, *L*, *F*, *J*, *H*, and *T* are the absolute difference matrices of GDP per capita, proportion of total tourism revenue in GDP, number of tourists as a percentage of residents, number of university students per 100,000 people, proportion of investment in environmental pollution control in GDP, and turnover of passenger traffic, respectively.

With UCINET software, the QAP regression analysis is carried out with the number of random permutations set at 5000. Table 5 displays the regression outcomes. With an adjusted R^2^ of 0.188, the regression equation describes 18.8% of the structure of the TES network. R^2^ values throughout earlier studies ranged from 12.5% to 40.3% [40,41]. This study’s R^2^ score is moderate, indicating its explanatory power.

The regression coefficient of geographical proximity is significantly positive (*p* < 0.01). This finding suggests that the closer the geographical proximity, the easier it is to build the spatial correlation link of TES. This positive promoting effect is mainly because geographical proximity can reduce the cost of component flow and information transmission and help realize the interprovincial sharing of TES.

The economic development level regression coefficient is considerably negative (*p* < 0.05). This finding suggests that the less the disparity in economic development, the more likely it is that similar ecological cognition and ecological management modes will emerge, resulting in similar ecological governance needs. Furthermore, due to market mechanism activity, factors of production are more likely to circulate among areas of similar economic development levels. Therefore, provinces with comparable economic development levels are easily correlated spatially for TES.

Tourism industry dependence affects TES in a significantly negative way (*p* < 0.05). Obviously, provinces with similar tourism industry dependence have more similar plans and goals for tourism industry development. They are willing to learn from each other’s tourism development models and tourism ecological protection measures and have closer information technology exchanges to strengthen the links of TES.

Transportation accessibility has a significantly negative impact on TES (*p* < 0.01). Thus, the smaller the disparity in transportation accessibility is, the more conducive it is to breaking the barrier of geographical distance, accelerating the exchange of the tourism economy, technology, and talent between regions, promoting learning and cooperation, and finally building a close spatial network.

The regression coefficients of the tourism load level, investment in environmental governance, and educational attainment are all significantly negative (*p* < 0.05). This result indicates that a smaller difference in the tourism load level, investment in environmental governance, and educational attainment means that human activities’ strain on the tourism ecosystem, people’s responses to changes in the tourism ecosystem, and the tourism ecosystem’s cognitive structure all share some characteristics. These similarities will promote the convergence of interprovincial TES management modes, and the convergence will help to establish close contacts between interprovincial TES.

## 4. Discussion

In this paper, SNA and QAP are used to explore the spatial network structure of TES and its influencing factors. Although 31 provinces in China are taken as examples, the framework of this study is still applicable to relevant studies in other parts of the world. The policy suggestions can provide a Chinese solution to promote the sustainable development of tourism in developing countries such as India and Thailand. 

The “east > central > west” distribution pattern can be seen in the strength of connections of TES, which is affected by geographical location, economic development level, and transportation accessibility. The TES connection has broken through the limitation of traditional geographical proximity and presents complex network structure characteristics. The overall spatial correlation network has good accessibility and distinct hierarchy, but the spatial linkage strength needs to be enhanced. Studies on ecological and environmental issues show that there is a significant spatial correlation between CO_2_ emissions of the global economy [42] and carbon emissions of the United States [43], which confirms the reliability of the research results in this paper.

The network of TES has a clear “core-edge” structure. The spatial correlation network is centered on the provinces of Jiangsu, Guangdong, Shandong, Zhejiang, and Henan, which act as crucial intermediary bridges. These provinces have distinct geographical locations, economic advantages, and easy access to transportation. Previous studies have shown that economic advantages have an important impact on TES. Regions with tourism economic development and sufficient capital have a higher level of TES and a stronger ability to absorb tourism production factors [1]. Guangxi, Shanghai, and Anhui are at the network’s periphery, with less spatial correlation with other provinces, which is closely related to their marginal geographical location and high pressure on the tourism ecosystem caused by a dense population. This finding is consistent with the result of individual network characteristics of forest ecological security [39].

The TES spatial network is separated into four segments: net spillover, agent, bidirectional spillover, and net benefit. Plate IV benefits greatly from Plates I and III, and the “siphon effect” is obvious. The clear spillover of TES in Plate I is not favorable to coordinated and sustainable tourism growth. As summarized by Liu et al. in their study [44], the clustering characteristics among network members are significant, the spatial correlation within the plates is relatively loose, there are spillover effects and synergistic effects between plates, and the polarization phenomenon of individual members is significant. 

The results of QAP analysis show that the differences in economic development level, tourism industry dependence, tourism load level, educational attainment, investment in environmental governance, and transportation accessibility all have a negative driving effect on the spatial network of TES, whereas geographic proximity has a positive driving effect. In other words, it is easier to form TES correlations between regions with similar economic development levels, tourism industry dependence, tourism load level, educational attainment, investment in environmental governance, and transportation accessibility. This is similar to the influencing factors of the provincial tourism eco-efficiency network [45].

It is worth noting that there are differences between the index system and influencing factors: on the one hand, the evaluation index is the component factor of TES. The TES index is calculated by the evaluation index in the form of absolute attribute data, which represents the development level of regional TES. On the other hand, the indicators of influencing factors represent interprovincial differences in economic, social, and environmental aspects. Its explanatory variable is the spatial correlation matrix of TES established by the modified gravity model. Based on QAP regression analysis, the model system of influencing factors constructed is used to explain inter-provincial differences in influencing factors on the TES networks. Therefore, both the establishment of the index system and the selection of influencing factors can effectively achieve the research purpose.

Unavoidably, there are some shortcomings. Regarding data availability, this work relied heavily on government statistics from primarily single-source channels. In the future, big data such as satellite remote sensing image data, pollution monitoring station data, tourism flows, and tourism enterprises can be applied to optimize the selection of indices for TES. In addition, this study takes provinces as its research units, and they are relatively macroscopic. If the research units are refined to cities or urban agglomerations, the spatial network description will be more accurate. Future research and optimization efforts should focus on these areas.

## 5. Conclusions

This paper has explored the spatial structure characteristics and influencing factors of TES in 31 Chinese provinces from the perspective of networks. The main research conclusions are as follows: (1) the spatial correlation network of provincial TES in China is increasingly close, but the network structure is loose and hierarchical. (2) The core–edge characteristics of individual network structures are clear, and the network role and status of provinces are closely related to their geographical location, economy, tourism resources, and transportation. (3) The TES networks can be divided into four parts: “net spillover”, “agent”, “bidirectional spillover” and “net benefit”. The internal connection of each plate is loose, and the spillover effect between plates is significant. (4) Geographical proximity, and the differences in economic development level, tourism industry dependence, tourism load level, educational attainment, investment in environmental governance, and transportation accessibility affect TES networks. 

Based on the research conclusion, the study makes policy recommendations for coordinating the development of regional TES. First, throughout the process of TES governance, we should not only concentrate on the quantity of TES but also the strength and stability of spatial correlation in different locations and realize the need to develop regional collaboration and coordination mechanisms. Second, the main participants or critical nodes in the construction of the target network are identified based on this “core-edge” structure and interactive relationship in the TES network. They could be used as catalysts for the exchange of tourism information resources and then become transmitters enhancing the interaction of TES. Third, the establishment of a spatial correlation network of TES is the result of multiple variables working together. Accelerating the transition of the economic development mode, advocating for green tourism development, boosting the monitoring of tourism environmental carrying capacity, and coordinating tourism development or pollution control investment are all important. Importantly, talent development and introduction, as well as economic, human, and technological exchanges and collaboration, should be prioritized to narrow regional gaps in TES and improve the overall level.

## Figures and Tables

**Table 1:** Evaluation index system for TES.

System Layer	Index Layer	Index Meaning	Unit	Weight	Reference
Drive (D)	GDP per capita	Socioeconomic development	CNY 10,000	0.0086	[1,20]
	Tertiary sector growth rate		%	0.0122	
	Urbanization rate		%	0.0127	
	Visitor growth rate		%	0.0227	
	Natural population growth rate	Tourism economy development	%	0.0115	
	Tourism revenue growth rate		%	0.0150	
Pressure (P)	Tourism economic density	Intensity of the combined pressure of tourism on the ecosystem	CNY 100,000,000/100,000 km^2^	0.0042	[1,21,30]
	Population density		per/hm^2^	0.0133	
	Visitor density		per/km^2^	0.0042	
	Total industrial wastewater	Negative impact of tourism economic development on ecosystem	10,000 tons	0.0080	
	Industrial SO_2_ emissions		10,000 tons	0.0097	
	Industrial solid waste		10,000 tons	0.0088	
	Amount of domestic waste removed		10,000 tons	0.0054	
State (S)	Domestic tourism revenue	Profitability of the tourism market	CNY 100,000,000	0.0287	[1,20,21]
	International tourism revenue		USD 100,000	0.0676	
	Ratio of visitor arrivals to the resident population	Provision of services for tourists	/	0.0089	
	Number of star-rated hotels		Individual	0.0108	
	Number of travel agencies		Individual	0.0136	
	Landscaped area	Quality of the living environment in the tourism destination	hm	0.0406	
	Public green space per capita		m^2^	0.0132	
	Forest coverage rate		%	0.0246	
Impact(I)	Share of tertiary sector output	Macro environment of tourism development	%	0.2053	[3,4,21]
	Tourism revenue index	Economic contribution rate of tourism	%	0.0260	
	Tourism income per capita		CNY	0.0225	
	Number of employees in the accommodation and catering industry	Reception capacity of the tourism destination	CNY 10,000	0.0526	
	Integrated utilization rate of industrial solid waste	Transformation and recycling of solid wastes in tourism destinations	%	0.0140	
Response (R)	Fiscal expenditure as a percentage of GDP	Driving force of tourism ecological environment investment	%	0.0438	[30,31]
	Environmental pollution control as a percentage of GDP		%	0.2622	
	Number of university students per 100,000 people	Tourism long-term development of talent supply	per	0.0142	
	Domestic sewage treatment rate	Local capacity for pollution control and resource recycling	per	0.0112	
	Domestic waste harmless disposal rate		%	0.0038	

**Table 2:** Centrality of the spatial association network of TES.

Rank	Province	Point Centrality			Betweenness Centrality	Closeness Centrality
		In-Degree	Out-Degree	Point		
1	Jiangsu	28	5	93.333	28.388	93.750
2	Guangdong	23	7	76.667	16.590	78.947
3	Shandong	20	6	66.667	8.854	68.182
4	Zhejiang	18	4	63.333	9.003	73.171
5	Henan	15	10	56.667	4.599	69.767
6	Sichuan	9	10	36.667	1.292	61.224
7	Ningxia	7	9	33.333	1.117	60.000
8	Shaanxi	5	9	30.000	0.300	58.824
9	Qinghai	4	8	26.667	0.234	57.692
10	Gansu	4	8	26.667	0.234	57.692
11	Hebei	6	8	26.667	0.574	57.692
12	Beijing	7	6	26.667	0.675	57.692
13	Guizhou	3	7	23.333	0.623	56.604
14	Jiangxi	6	6	23.333	0.750	56.604
15	Inner Mongolia	4	7	23.333	0.395	56.604
16	Liaoning	2	7	23.333	0.193	56.604
17	Jilin	1	7	23.333	0.338	56.604
18	Heilongjiang	1	7	23.333	0.338	56.604
19	Xinjiang	0	6	20.000	0.234	55.556
20	Tibet	1	6	20.000	0.234	55.556
21	Chongqing	2	6	20.000	0.234	55.556
22	Hubei	2	6	20.000	0.172	55.556
23	Shanxi	4	6	20.000	0.210	55.556
24	Yunnan	1	5	16.667	0.174	54.545
25	Hainan	4	2	16.667	0.226	53.571
26	Hunan	3	5	16.667	0.207	54.545
27	Fujian	1	5	16.667	0.213	54.545
28	Tianjin	4	5	16.667	0.101	54.545
29	Guangxi	1	3	10.000	0.016	51.724
30	Shanghai	2	3	10.000	0.035	51.724
31	Anhui	2	1	6.667	0.000	50.000
Mean	-	6.129	6.129	29.462	2.469	59.266

**Table 3:** Spillover impact of plates.

Plate	Reception		Spillover		Expected Ratio/%	Actual Ratio/%	Role of the Plate
	Internal	External	Internal	External			
Plate I	20	29	20	91	50	18.018	Net spillover
Plate II	8	13	8	23	23.333	25.806	Agent
Plate III	3	48	3	29	10	9.375	Bidirectional spillover
Plate IV	2	67	2	14	6.667	12.500	Net benefit

**Table 4:** Density matrix and image matrix.

Plate	Density Matrix				Image Matrix			
	Plate I	Plate II	Plate III	Plate IV	Plate I	Plate II	Plate III	Plate IV
Plate I	0.083	0.008	0.719	0.917	0	0	1	1
Plate II	0.016	0.143	0.063	0.792	0	0	0	1
Plate III	0.391	0	0.250	0.333	1	0	1	1
Plate IV	0.042	0.5	0	0.333	0	1	0	1

**Table 5:** The results of QAP correlation and regression analysis.

Independent Variable	QAP Correlation Analysis		QAP Regression Analysis	
	Correlation Coefficient	*p* Value	Regression Coefficient	*p* Value
*W*	0.291	0.000 ***	0.193	0.000 ***
*E*	−0.16	0.005 ***	−0.096	0.022 **
*L*	−0.123	0.023 **	−0.057	0.046 **
*F*	−0.085	0.095 *	−0.044	0.041 **
*J*	−0.064	0.061 *	−0.080	0.034 **
*H*	−0.053	0.012 **	−0.008	0.014 **
*T*	−0.192	0.000 ***	−0.161	0.000 ***
*R* ^2^			0.195	0.000 ***
*Adj*-*R*^2^			0.188	0.000 ***

*, **, and *** represent significance at the 10%, 5%, and 1% levels, respectively.

## Data Availability

The data presented in this study are available on request from the corresponding author.
